# Timing and Distribution of Mitotic Activity in the Retina During Precocial and Altricial Modes of Avian Development

**DOI:** 10.3389/fnins.2022.853544

**Published:** 2022-05-09

**Authors:** Guadalupe Álvarez-Hernán, José Antonio de Mera-Rodríguez, Ismael Hernández-Núñez, Abel Acedo, Alfonso Marzal, Yolanda Gañán, Gervasio Martín-Partido, Joaquín Rodríguez-León, Javier Francisco-Morcillo

**Affiliations:** ^1^Departamento de Anatomía, Biología Celular y Zoología, Facultad de Ciencias, Universidad de Extremadura, Badajoz, Spain; ^2^Departamento de Anatomía, Biología Celular y Zoología, Facultad de Ciencias, Universidad de Extremadura, Badajoz, Spain; ^3^Departamento de Anatomía, Biología Celular y Zoología, Facultad de Medicina y Ciencias de la Salud, Universidad de Extremadura, Badajoz, Spain

**Keywords:** altricial, apical mitoses, bird retina, embryo, horizontal cells, immunohistochemistry, non-apical mitoses, precocial

## Abstract

During development of the vertebrate retina, mitotic activity is defined as apical when is located at the external surface of the neuroepithelium or as non-apical when is found in more internal regions. Apical mitoses give rise to all retinal cell types. Non-apical mitoses are linked to committed horizontal cell precursors that subsequently migrate vitreo-sclerally, reaching their final position in the outer surface of the inner nuclear layer, where they differentiate. Previous studies have suggested differences in the timing of retinal maturation between altricial and precocial bird species. In the present study we analyze qualitatively and quantitatively the mitotic activity in the developing retina of an altricial (zebra finch, *Taeniopygia guttata*) and a precocial (Japanese quail, *Coturnix coturnix*) bird species. We found that pHisH3-immunoreactive apical and non-apical mitoses were abundant in the *T. guttata* retina at the hatching stage. In contrast, pHisH3 immunoreactivity almost disappeared from the quail retina at the embryonic day 10 (E10). Furthermore, we also found that the onset of the appearance of non-apical mitoses occurred at later stages in the altricial bird species than in the precocial one. The disappearance of apical mitoses and the spatiotemporal distribution of non-apical mitoses followed central to peripheral and dorsal to ventral gradients, similar to gradients of cell differentiation described in the retina of birds. Therefore, these results suggest that retinal neurogenesis is active at the hatching stage in *T. guttata*, and that horizontal cell differentiation is delayed in the altricial bird species compared to the precocial one. Together, this study reveals important insights into the timing differences that regulate bird retinal maturation and provides a better understanding of the evolution of avian altriciality and precociality.

## Introduction

Developmental events in the retina, such as cell proliferation, cell migration, and cell differentiation occur in a precise and predetermined order (Amini et al., 2018). The retina develops from an undifferentiated neuroepithelium to a laminated structure in which neurons and glial cells are arranged in different layers (Hoon et al., 2014; Souza de Campos et al., 2020). Pseudostratified neuroepithelia consists of elongated retinal progenitor cells that are polarized along their apicobasal axis, undergoing apical (also known as “ventricular” or “distal”) division that give rise to different cell types that migrate in a scleral to vitreal manner until they reach their final position (Lee and Norden, 2013). A less numerous population of dividing cells is also found in basal regions of the presumptive retina (also known as “non-apical, “non-ventricular,” “extra-ventricular,” “proximal,” “vitreal” mitoses). This non-apical mitotic activity has been found in fish (Godinho et al., 2007; Bejarano-Escobar et al., 2012; Weber et al., 2014; Engerer et al., 2017), birds (Edqvist and Hallböök, 2004; Boije et al., 2009, 2016; Shirazi Fard et al., 2013, 2014a,2014b; Álvarez-Hernán et al., 2018), and mammals (Rapaport et al., 1985; Robinson et al., 1985; Sharma and Ehinger, 1997) and has been linked to horizontal cell differentiation. After this division in the vitreal side of the neuroepithelium, horizontal precursors migrate vitreo-sclerally to their final laminar position in the external region of the inner nuclear layer (INL) (for a review, see Boije et al., 2016). During early stages of retinogenesis, uniform mitotic activity is found over the scleral surface of the retina, but mitotic figures disappear following the central to peripheral and dorsal to ventral gradients of cell differentiation (Edqvist and Hallböök, 2004).

In nature, there is a wide spectrum of avian hatchlings from altricial to precocial extreme (Starck and Ricklefs, 1998). At hatching, precocial birds are born covered in feathers with their eyes open, have locomotion, and can feed themselves soon after hatching. On the contrary, altricial birds are born nearly naked with their eyes closed, display no locomotor activity and are exclusively fed by parents. Then, the functionality of sense organs at hatching varies across the altricial-precocial spectrum. Previous data suggest that the pattern of eye pigmentation and retinal maturation differs between precocial and altricial bird species (Fischer and Reh, 2000; Rojas et al., 2007; Olea and Sandoval, 2012; Murray et al., 2013; Olea et al., 2016; Álvarez-Hernán et al., 2018, 2020, 2021a,2021b). The higher sensory and locomotor capabilities of precocial bird species at birth might indicate higher prenatal neurogenesis and retinal maturation, while ontogenetic retinal events extend into the early postnatal life in altricial birds (Da Costa Calaza and Gardino, 2010; Vergara and Canto-Soler, 2012; Wisely et al., 2017; Álvarez-Hernán et al., 2018, 2020, 2021a,2021b,2021c; de Mera-Rodríguez et al., 2019, 2021; Ghinia-Tegla et al., 2021).

The chicken (Fischer and Reh, 2000; Fischer et al., 2008; de Mera-Rodríguez et al., 2019, 2021; Álvarez-Hernán et al., 2021c) and the zebra finch (*Taeniopygia guttata*, Vieillot 1817) (Álvarez-Hernán et al., 2018, 2020, 2021a,2021b) constitute excellent models in which to study patterns of visual system development from a precocial-altricial perspective. The Japanese quail (*Coturnix coturnix*, Linnaeus 1758) is a notable avian model species that belongs to the order of Galliformes. It has been widely used by researchers to study several aspects of visual development, such as the entry of microglia in the developing retina (Sánchez-López et al., 2004; Martín-Estebané et al., 2017) and the coincidence between microglia immigration and developmental cell death (Marín-Teva et al., 1999). However, little is known about cell proliferation and differentiation during retinogenesis in this precocial bird species.

Studies on detailed direct comparison of different aspects of retinal development between precocial and altricial bird species are sparse. The aim of the present study is to compare patterns of retinal development, specifically mitotic activity, in precocial and altricial bird species. The arised results could provide new insights into the timing of cell proliferation and cell differentiation in the bird retina and a greater understanding of the evolutionary mechanisms involved in the process of speciation within the precocial-altricial spectrum.

## Materials and Methods

### Animal and Tissue Processing

All animals manipulations have been performed in accordance with the National and European legislation (Spanish Royal Decree RD53/2013 and EU Directive 86/609/CEE as modified by 2003/65/CE, respectively). Experimental protocols were approved by the Bioethics Committee for Animal Experimentation of the University of Extremadura (Ref 264/2019). Fertilized *T. guttata* (*n* = 63) and *C. coturnix* (*n* = 45) eggs were placed within a rotating egg incubator designed for chicken eggs (Masallés S.A.) and were maintained at 37.5 ± 1°C and 80–90% humidity. Eggs were placed in a small padded dish on the rotating bars of the incubator. They roll within the dish preventing embryo adhesion to the shell. A total of 34 *T. guttata* embryos and 34 embryos of *C. coturnix* and 2 newly hatched animals were used in the present study (Table 1). The oldest embryos of *T. guttata* were kindly provided by Dr. Michiel Vellema, from the University of Utrech, Netherlands. Zebra finch embryos were staged according to Murray et al. (2013). The degree of development of the quail embryos was estimated according to the stages established by Ainsworth et al. (2010). Embryos were fixed with paraformaldehyde (PFA) 4% in phosphate-buffered solution (PBS) (0.1M, pH 7.4) overnight at 4°C. For immunohistochemical techniques, embryos were immersed in a cryoprotective solution (15% sucrose in PBS) overnight at 4°C, soaked in embedding medium and frozen for 5 min in isopentane cooled to −70°C by dry ice, and then stored at −80°C until use. Cryosections of 20 μm were obtained in a cryostat microtome (Leica CM 1900), thaw-mounted on SuperFrost Plus slides, air-dried and stored at −20°C.

**TABLE 1:** *T. guttata* and *C. coturnix* embryos and hatchlings used in the present study.

Embryonic day/Stage (Murray et al., 2013)	Number of *T. guttata* individuals	Embryonic day/Stage (Ainsworth et al., 2010)	Number of *C. coturnix* individuals
E5/St27	6	E5/St27	6
E6/St30	5	E6/St30	6
E7.5-8/St34	6	E7.5-8/St34	6
E8-9/St38	6	E8-9/St36-37	6
E10/St41	6	E10/St38	5
E13/St45	5	E13/St42	5
E17/P0/St46	1	E17/P0/St46	1

### Immunohistochemistry

Sections were subjected to an antigen retrieval process with citrate buffer (pH 6.0) at 90°C during 30 min. Sections were chilled at room temperature for 20 min. Slides were washed one time in 0.1% Triton-X-100 in PBS (PBS-T) and two times in 0.2% gelatin, 0.25% Triton-X-100 in PBS (PBS-G-T) and then pre-blocked in 0.2% gelatin, 0.25% Triton-X-100, Lys 0.1 M in PBS (PBS-G-T-L) for 1 h. Sections were incubated with the primary antibody over night at RT in a humidified chamber:

1. rabbit anti-phisH3 (1:200, Millipore, 06-570) polyclonal antibody, that identifies cells in late G2/M-phase in the chicken (Boije et al., 2009; Shirazi Fard et al., 2013, 2014a,2014b; Blixt et al., 2018) and zebra finch retina (Álvarez-Hernán et al., 2018, 2020, 2021a).
2. rabbit anti-Prox1 polyclonal antibody (1:200, Millipore, 07634), that identifies a transcription factor that is present in all horizontal cells in the chicken (Edqvist et al., 2008; Boije et al., 2009) and in the zebra finch (Álvarez-Hernán et al., 2020).
3. mouse anti-Islet1 (Isl1) monoclonal antibody (1:200, Developmental Studies Hybridoma Bank, 39.4D5), that labels a subpopulation of horizontal cells in the retina of the chicken (Edqvist et al., 2008; Boije et al., 2009; Bejarano-Escobar et al., 2015) and zebra finch (Álvarez-Hernán et al., 2020).

After incubation with the primary antibody, slides were washed several times in PBS-T and PBS-G-T and incubated with Alexa Fluor 488 goat anti-mouse IgG antibody (1:200, Molecular Probes, A11029), Alexa Fluor 488 goat anti-rabbit IgG antibody (1:200, Molecular Probes, A11008) and Alexa Fluor 594 goat anti-rabbit IgG antibody (1:200, Molecular Probes, A11037) during 2 h at RT in a humidified chamber in darkness. Sections were washed two times in PBS-T and one in PBS-G-T in darkness. The tissue was incubated for 10 min with DAPI at RT and then washed three times in PBS. Slides were mounted with Mowiol (MKBD8495V, SIGMA).

### Image Acquisition and Processing

Immunofluorescence sections were observed with a bright field and epifluorescence Nikon Eclipse 80i microscope and photographed using an ultra-high-definition Nikon DXM1200F digital camera. Images were processed with Adobe Photoshop CS4.

### Quantification of Apical and Non-apical Mitosis

**FIGURE 1:**
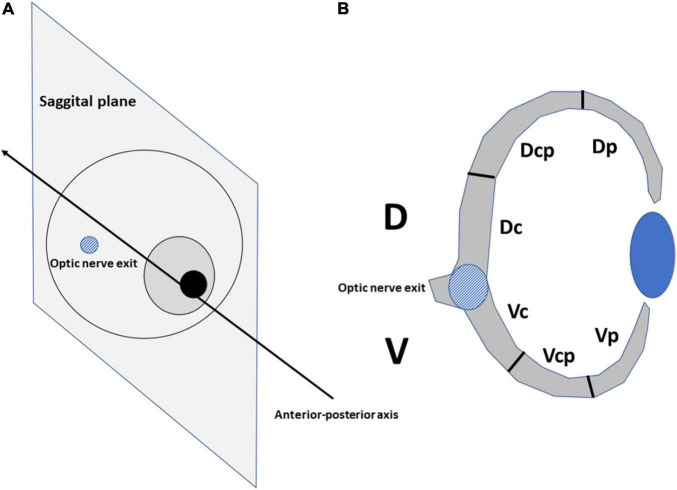
The eyes of bird embryos were cut at the level of the optic nerve exit in the sagittal plane **(A)**. The dorsal (D) and ventral (V) halves of the retina were divided into sectors into three equally large sectors by measuring the length of the inner limiting membrane **(B)**: dorso-central (Dc), dorso-centro-peripheral (Dcp), dorso-peripheral (Dp), ventro-central (Vc), ventro-centro-peripheral (Vcp), and ventro-peripheral (Vp).

The eyes of the embryos were sectioned parallel to the sagittal plane (Howland and Howland, 2008), allowing the dorsal (D) and ventral (V) retinal regions to be distinguished when the optic nerve was sectioned (Figure 1). Sections were stained with the anti-pHisH3 antibody in combination with anti-Prox1 and anti-Isl1 antibodies. The D and V regions were divided into three equally large sectors by measuring the length of the inner limiting membrane (ILM): dorso-central (Dc-near the optic nerve exit), dorso-centro-peripheral (Dcp), Dorso-peripheral [Dp-near the ciliary marginal zone (CMZ)], Ventro-central (Vc), Ventro-centro-peripheral (Vcp), and Ventro-peripheral (Vp) (Figure 1). We captured fluorescence images of the entire retina using a 20x objective. As development progresses, a higher number of 20x images were included in each sector. The length of the ILM and the surface area of the retina in digital micrographs was measured using the ImageJ free open-source software package (^1^ accessed on 28 January 2021). The density profiles were expressed as the mean ± sem of the number of pHisH3-positive mitoses per square millimeter (mitosis/mm^2^). Statistical analyses and the graphs were performed with the R programming language. To analyze if there were significative differences, the Mann–Whitney *U* test was used. Differences between groups were considered as significant when *P* < 0.05 and highly significant when *P* < 0.001.

## Results

### Developmental Events and Immunochemical Profiles in the Quail Retina

Toluidine blue-stained semi-thin sections in the undifferentiated quail retina showed abundant mitoses mainly localized in the scleral surface of the neuroblastic layer (NbL), but sparse non-apical mitoses were also observed. We also evaluated the specificity of primary antibodies in the quail retina by comparison with published examples of labeling results and immunoassays in other bird species, and we found similar expression patterns for pHisH3, Prox1, and Isl1 antibodies (see Supplementary Figure 1). None of the observed staining was due to non-specific binding of secondary antibody or autofluorescence in the fixed tissues because sections labeled with secondary antibodies alone were devoid of fluorescence. Therefore, the location of mitotic activity and the immunohistochemical patterns observed in the developing quail retina were similar to that described in the retina of other bird species.

#### Identification of Mitotic Figures in the Retinal Tissue in Precocial and Altricial Bird Species at Hatching Stage

**FIGURE 2:**
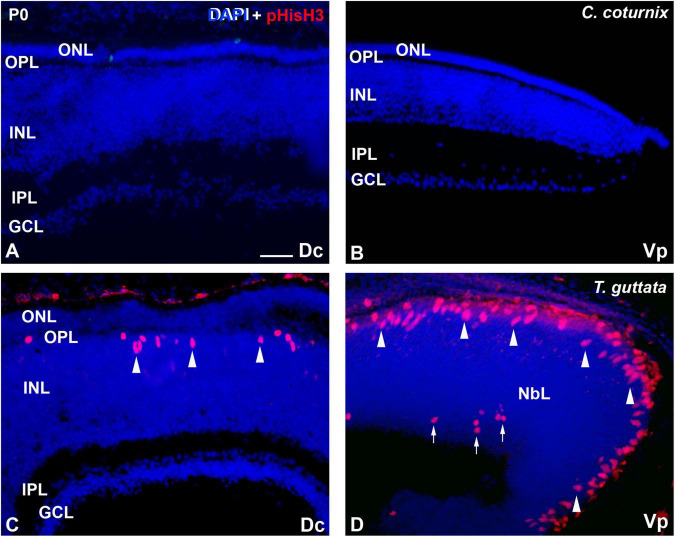
Patterns of pHisH3 immunoreactivity Dc **(A,C)** and Vp regions **(B,D)** of the *C. coturnix* **(A,B)** and *T. guttata* **(C,D)** retina at hatching. Cryosections were counterstained with DAPI. **(A,B)** Immunoreactive mitotic figures were totally absent from the *C. coturnix* retina. **(C,D)** pHisH3-immunoreactive mitoses were mainly located in the vitreal surface (arrowheads) in the *T. guttata* retina. Furthermore, non-apical mitoses were also found in the Vp sector (arrows). *Abbreviations: GCL, ganglion cell layer; INL, inner nuclear layer; IPL, inner plexiform layer; NbL, Neuroblastic layer; ONL, outer nuclear layer; OPL, outer plexiform layer. Scale bar:* 25 μm.

The *C. coturnix* retina did not show immunoreactivity against pHisH3 neither in central (Figure 2A) nor peripheral regions (Figure 2B). In contrast, the *T. guttata* retina clearly showed abundant pHisH3-immunoreactive mitotic figures in apical position in both the central (Figure 2C) and peripheral retina (Figure 2D). Furthermore, some non-apical mitoses were also distinguished in the peripheral retina (Figure 2D).

#### Temporospatial Distribution of Mitotic Activity and Horizontal Cell Markers in the Embryonic Retina in Altricial Bird Species

**FIGURE 3:**
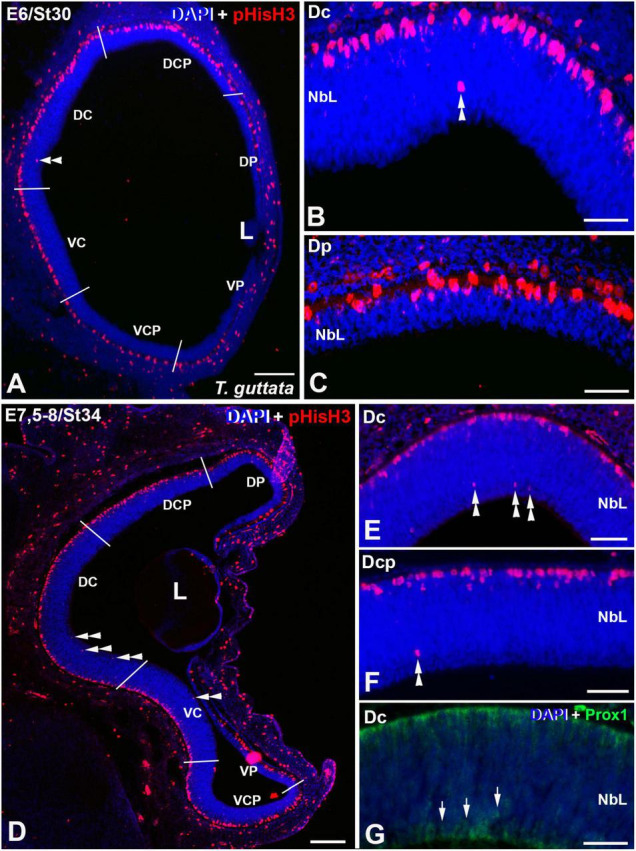
Expression patterns of pHisH3 **(A–F)** and Prox1 **(G)** in retinal cryosections of *T. guttata* at E6 (St30) **(A–C)** and E7.5-8 (St34) **(D–F)**. Cryosections were counterstained with DAPI. **(A–C)** The first non-apical mitosis were detected at E6 in the Dc sector (double arrowheads in A,B). The Dp sector presented an accumulation of pHisH3-immunoreactive mitoses in the apical region of the NbL. **(D–G)** At E7.5-8, the incidence of pHisH3 positive non-apical mitoses increased in Dc and Dcp sectors [double arrowheads in panels **(D–F)**]. **(G)** Prox1-positive nuclei were detected in close relationship with non-apical mitoses (arrows). *Abbreviations: L, lens; NbL, Neuroblastic layer. Scale bars:* 100 μm in panels **(A,D)**; 25 μm in panels **(B,C)**; 50 μm in panels **(E–G)**.

Mitotic activity was intense and homogeneously distributed in the apical region of the retinal NbL during early stages of development in *T. guttata* (see Álvarez-Hernán et al., 2020). At St30 (E6) the first non-apical mitoses were detected in the Dc sector, but the ventricular side contained most of the pHisH3 mitotic figures (Figures 3A,B, 6A,B). At this stage, non-apical mitoses were absent in peripheral sectors (Figure 3C). No immunoreactivity against Prox1 was detected in the NbL by this stage (not shown). At St34 (E7.5-8), cell division was intense in the ventricular region of the NbL and the density of non-apical pHisH3-immunoreactive mitoses increased mainly in the D region of the retina, but also in the Vc sector (Figures 3D–F, 6A,B). By this stage, Prox1 immunoreactivity was first detected in the inner region of the NbL (Figure 3G), coinciding temporospatially with the mitotic activity detected in the non-apical region (Figures 3D–G).

**FIGURE 4:**
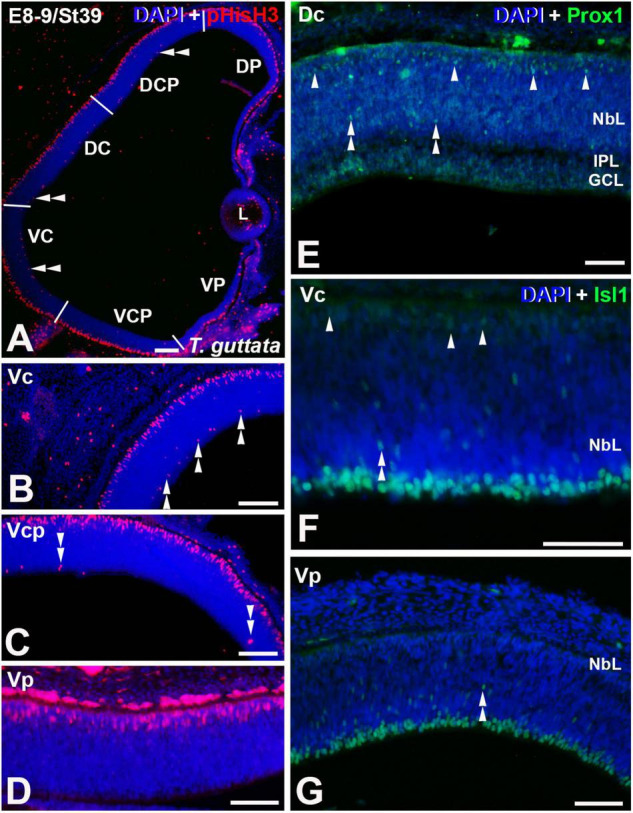
Expression patterns of pHisH3 **(A–D)**, Prox1 **(E)**, and Isl1 **(F,G)** in retinal cryosections of *T. guttata* at E8-9 (St39). Cryosections were counterstained with DAPI. **(A–D)** Non-apical mitoses were mainly detected in Dp, Vc, and Vcp sectors [double arrowheads in panels **(A–C)**]. **(E)** Retinal lamination was almost complete in the Dc sector and Prox1 immunoreactivity was mainly restricted to the region of the presumptive horizontal cell layer (arrowheads). Sparse Prox1-immunoreactive nuclei were detected in more internal regions (double arrowheads). **(F,G)** Isl1-immunoreactive migrating neuroblasts were dispersed throughout the NbL (double arrowheads). Isl1-immunoreactive presumptive horizontal cells were detected in the Vc region [arrowheads in panel **(F)**]. *Abbreviations: GCL, ganglion cell layer; IPL, inner plexiform layer; NbL, Neuroblastic layer. Scale bars:* 50 μm.

**FIGURE 5:**
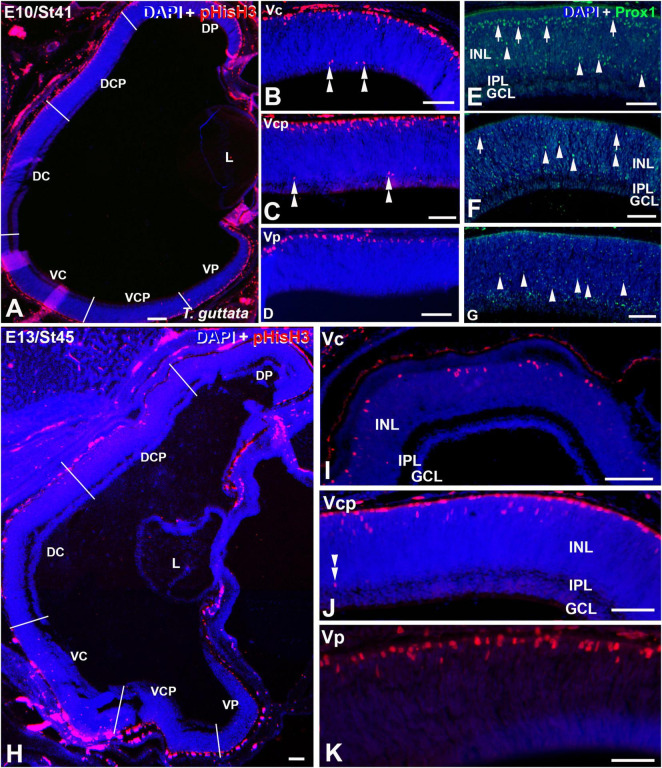
Retinal cryosections of *T. guttata* at E10 (St41) **(A–G)** and E13 (St45) **(H–K)** immunolabelled with antibodies against pHisH3 **(A–D,H–K)** and Prox1 **(E–G)**. Cryosections were counterstained with DAPI. **(A–D)** Non-apical mitoses were restricted to the V retinal half (double arrowheads), mainly to the Vc and Vcp sectors **(B,C)**. Prox1 immunoreactivity located in the presumptive horizontal cell layer progressively decreased from the Vc to the Vp sector [arrows in panels **(E,F)**]. In contrast, Prox1 immunoreactivity in more internal regions progressively increased from the Vc to the Vp region [arrowheads in panels **(E–G)**]. **(H–K)** Non-apical mitoses were mainly distributed in the Vc and Vcp sectors (double arrowheads). Notice that the pHisH3-immunoreactive figures located in the apical region of the E13 retina are less numerous than that observed at previous stages. *Abbreviations: GCL, ganglion cell layer; INL, inner nuclear layer; IPL, inner plexiform layer; NbL, Neuroblastic layer. Scale bars:* 50 μm in panels **(A–I)**; 25 μm in panels **(J,K)**.

**FIGURE 6:**
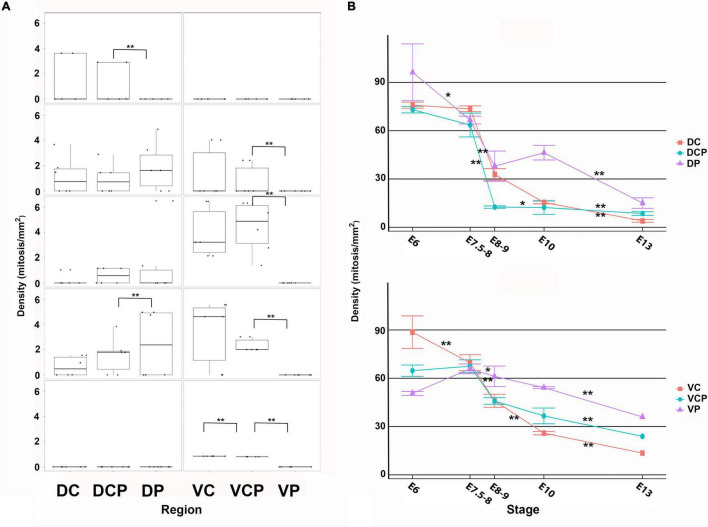
Density values non-apical **(A)** and apical **(B)** mitoses in the developing retina of *T. guttata.* For each retinal region, boxplots of all samples are shown with the average, median, interquartile, and outliers. Notice that the non-apical mitoses spread following a dorsal-to-ventral gradient **(A)**. Intense mitotic activity was detected in the apical region of the ventral retina at advanced stages of development. Data are expressed as means ± s.e.m. Statistical significance is indicated by asterisks (*p < 0.05, **p < 0.01).

At St39 (E8-9) the laminated structure of the retina could be distinguishable in the Dc sector (Figures 4A,E). At this stage, non-apical mitoses were mainly distributed in the Dp, Vc, and Vcp sectors (Figures 4A–D, 6A). Intense Prox1 immunoreactivity was found in the Dc sector in the presumptive horizontal cell layer (Figure 4E). However, Isl1 immunoreactive nuclei in the horizontal cell layer were sparse in the Vc sector (Figure 4F), and they were absent from the Vp sector (Figure 4G). The density of mitotic activity in the apical region diminished in the dorsal retina while is maintained or increased in the different sectors of the ventral retina (Figure 6B).

Non-apical mitoses were mainly detected in the Vc and Vcp sectors by E10-E13 (Figures 5A–D,H–K, 6A), coinciding with abundant Prox1-immunoreactive nuclei dispersed throughout de presumptive INL (Figure 5E,F), but they were almost absent from the Vp one (Figure 5G). Regarding pHisH3 immunoreactive apical mitoses (Figure 5B), they were present in all the sectors during the period analyzed in the present study. During last stages of development, the density values of non-apical mitoses in the ventral sectors were higher than those observed in the dorsal retina (Figure 6B).

At E13, non-apical mitoses were absent from the dorsal retina (Figures 5H, 6A) and they were mainly found in the VC and Vcp sectors (Figures 5I,J, 6A). High levels of pHisH3-immunoreactive elements were found in the ventral retina, while they were almost absent from the dorsal retina (Figures 5H, 6B).

#### Temporospatial Distribution of Mitotic Activity and Horizontal Cell Markers in the Embryonic Retina in Precocial Bird Species

**FIGURE 7:**
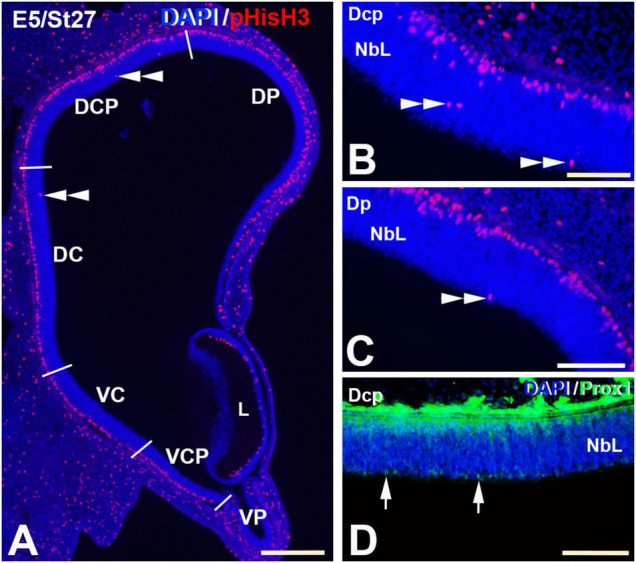
pHisH3 **(A–C)** and Prox1 **(D)** immunolabeling in the E5 (St27) *C. coturnix* retina. Cryosections were counterstained with DAPI. **(A–C)** Non-apical mitosis were restricted to the D retina, mainly to the Dc and Dcp sectors (double arrowheads). Apical mitoses were abundant in the entire retina. **(D)** Prox1-positive nuclei were detected in close relationship with non-apical mitoses (arrows). *Abbreviations: L, Lens; NbL, Neuroblastic layer. Scale bars:* 100 μm in panel **(A)**; 50 μm in panels **(B–D)**.

**FIGURE 8:**
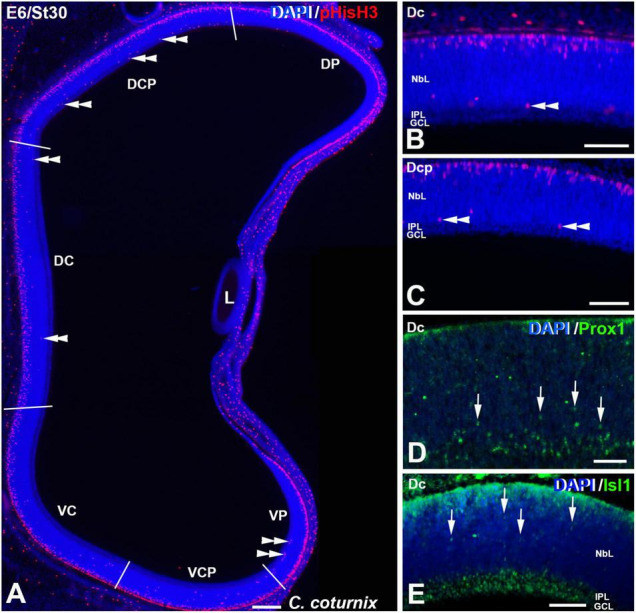
pHisH3 **(A–C)**, Prox1 **(D)** and Isl1 **(E)** immunoexpression in the *C. coturnix* retina at E6 (St30). Cryosections were counterstained with DAPI. **(A–C)** Non-apical mitoses were detected in all the sectors analyzed (double arrowheads). **(D,E)**. Abundant Prox1- (arrowheads in D) and Isl1- (arrowheads in E) immunoreactive nuclei of migrating neuroblasts were found dispersed throughout the NbL. *Abbreviations: GCL, ganglion cell layer; IPL, inner plexiform layer; NbL, Neuroblastic layer. Scale bars:* 100 μm in panel **(A)**; 50 μm in panels **(B–E)**.

**FIGURE 9:**
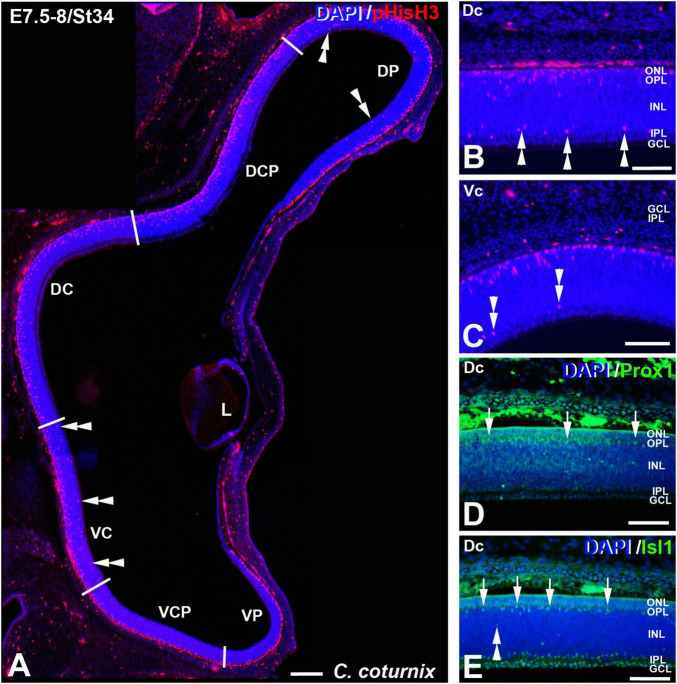
pHisH3 **(A–C)**, Prox1 **(D)**, and Isl1 **(E)** immunolabeling in the *C. coturnix* retina at E7.5-8 (St34). Cryosections were counterstained with DAPI. **(A–C)** Non-apical mitosis were mainly located in the D retina but also in the Vc sector (double arrowheads). **(D,E)** Abundant Prox1- and Isl1-immunoreactive horizontal cells were detected in the horizontal cell layer of the Dc sector (arrows). *Abbreviations: GCL, ganglion cell layer; INL, inner nuclear layer; IPL, inner plexiform layer; L, lens; ONL, outer nuclear layer. Scale bars:* 100 μm in panel **(A)**; 50 μm in panels **(B–E)**.

The St27 (E5) retina, the first stage analyzed in *C. coturnix*, showed that non-apical mitoses were mainly concentrated in the dorsal sectors of the NbL, but also in the Vc sector (Figures 7A–C, 11A). Prox1-immunoreactive nuclei were found in the inner region of the NbL, in close relationship with non-apical mitoses (Figure 7D). Between St30 (E6) and St34 (E7.5-8) higher density values of pHisH3 immunoreactive non-apical mitoses were found in the dorsal sectors (Figures 8A–C, 9A,B, 11A), but also in the ventral sectors (Figures 9C, 11A), coinciding topographically with Prox1-(Figures 8D, 9D) and Isl1 (Figures 8E, 9E) immunoreactive nuclei. At St34, many Prox1- and Isl1-immunoreactive nuclei were located in the presumptive horizontal cell layer (Figures 9D,E).

**FIGURE 10:**
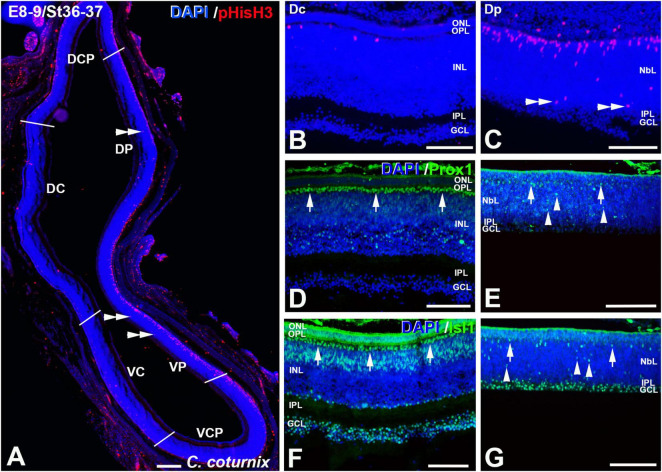
pHisH3 **(A–C)**, Prox1 **(D,E)**, and Isl1 **(F,G)** immunolabeling in the *C. coturnix* retina at E8-9 (St36-37). Cryosections were counterstained with DAPI. **(A–C)** Non-apical mitosis were mainly located in the Dp and Vp sectors (double arrowheads). Apical mitoses were scarce in the Dc **(B)**, Dcp, Vc, and Vcp sectors **(A)**. **(D–G)** Abundant Prox1- [arrows in panel (D)] and Isl1- [arrows in panel **(F)**] immunoreactive nuclei were detected in the horizontal cell layer in the Dc sector, but also in the amacrine cell layer **(D,F)** and in the GCL **(F)**. More immature staining patterns were found in the Dp sector, with sparse immunoreactive nuclei located in the presumptive horizontal cell layer [arrows in panels **(E,G)**] but also in more internal regions of the NbL [arrowheads in panels **(E,G)**]. *Abbreviations: GCL, ganglion cell layer; INL, inner nuclear layer; IPL, inner plexiform layer; NbL, Neuroblastic layer; ONL, outer nuclear layer. Scale bars:* 100 μm in panel **(A)**; 50 μm in panels **(B–G)**.

**FIGURE 11:**
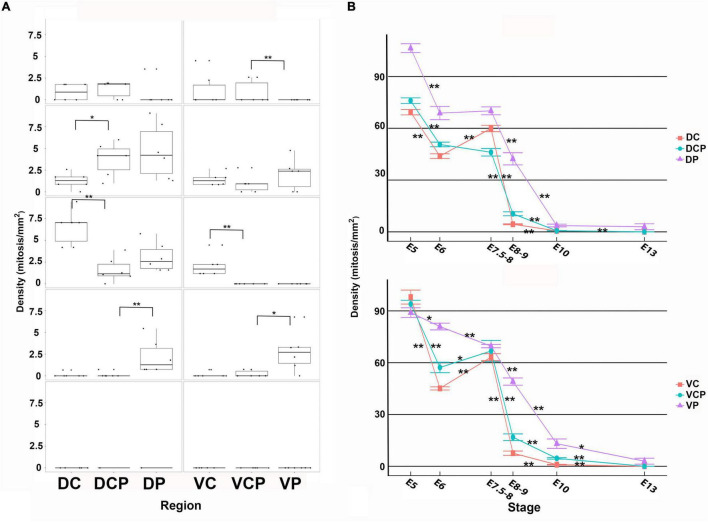
Density values non-apical **(A)** and apical **(B)** mitoses in the developing retina of *C. coturnix.* For each retinal region, boxplots of all samples are shown with the average, median, interquartile, and outliers. Notice that at early stages the non-apical mitoses were mainly concentrated in the dorsal retina and at E8-9, they were mainly restricted to the Dp and Vp sectors. From this stage onwards, non-apical mitoses disappeared from the entire retina. Apical mitoses were almost absent from E8-9 onward in the Dc, Dcp, Vc, and Vcp sectors. The density values were very low in the Dp and Vp sectors at E13. Data are expressed as means ± s.e.m. Statistical significance is indicated by asterisks (*p < 0.05, **p < 0.01).

At E8-9 non-apical mitoses almost disappeared from the Dc, Dcp, Vc, and Vcp sectors, but they were still detected in the Dp and Vp sectors (Figures 10A–C, 11A). Accordingly, while the density values of apical mitoses were higher at previous stages, at this stage decreased sharply in the Dc, Dcp, Vc, and Vcp sectors (Figure 11B). However, these density values remained high at this stage in the Dp and Vp sectors (Figure 11B). In the regions that were devoid of non-apical mitoses, the patterns of distribution of Prox1 and Isl1 in the horizontal cell layer were similar to that described in the mature chicken retina (Figures 10D,F; Edqvist et al., 2006; Bejarano-Escobar et al., 2015). In the Dp and Vp sectors, these transcription factors were detected only in a few nuclei located in the presumptive horizontal cell layer (Figures 10E,G). From this stage onward (E10-E13), non-apical mitoses disappeared from the entire retina (Figure 11B) and apical mitoses were sporadically observed in the Dp and Vp sectors (Figure 11B).

Therefore, non-apical divisions in the developing retina of birds spread following central to peripheral and dorsal to ventral gradients, and their appearance coincides chronotopographically with the onset of Prox1 immunoreactivity. When the non-apical divisions disappear, Prox1-immunoreactivity is located mainly in the nuclei of differentiated horizontal cells. Additionally, the disappearance of apical mitoses followed the same gradients described above. Finally, while abundant proliferative activity is detected in the retina of altricial birds at hatching, residual mitotic figures are only observed in the peripheral region of the precocial retina from E10 until the hatching stage (E16.5).

## Discussion

Our data demonstrated that the disappearance of apical mitoses and the spatio-temporal distribution of non-apical mitoses in the retina of altricial and precocial birds follows central to peripheral and dorsal to ventral gradients. Closely coincident temporal and spatial patterns of cell genesis have been described in the *G. gallus* (Prada et al., 1991; Francisco-Morcillo et al., 2005; Drenhaus et al., 2007; Bejarano-Escobar et al., 2015; de Mera-Rodríguez et al., 2019; Álvarez-Hernán et al., 2021c) and *T. guttata* retina (Álvarez-Hernán et al., 2018, 2020). Similar profiles of cell differentiation have been described in the retina of fish (Vecino et al., 1993; Candal et al., 2005, 2008; Bejarano-Escobar et al., 2014), reptiles (Francisco-Morcillo et al., 2006), and mammals (Young, 1985; Reese et al., 1996). The incidence of cell proliferation in the retina of newly hatched animals, the appearance and disappearance of mitotic figures, and the relationship between the spatio-temporal location of non-apical mitoses with the onset of appearance of horizontal cell markers will be discussed below under altricial-precocial spectrum.

### Mitotic Activity in the Retina During Development: Differences Between Altricial and Precocial Bird Species

#### Mitotic Activity in the Bird Retina at Hatching

Abundant mitotic figures were found in all the sectors analyzed in the present study in the *T. guttata* retina at hatching, in concordance with previous studies conducted in altricial bird species (Rojas et al., 2007; Álvarez-Hernán et al., 2018, 2020, 2021a). Other features of immaturity were described in the altricial bird retina of newly hatched animals, such as very thin plexiform layers, ganglion cell layer containing many thick cells, and photoreceptors showing poorly developed outer segments. Furthermore, ontogenetic events that are restricted to the embryonic retina in precocial bird species such as cell death (Cook et al., 1998; Marín-Teva et al., 1999; Francisco-Morcillo et al., 2014) are detected in the altricial bird retina during the first week of life (Álvarez-Hernán et al., 2021b). A high abundance of proliferative progenitors has been also described in the retina of altricial newly hatched fish (Evans and Browman, 2004; Bejarano-Escobar et al., 2014; Pavón-Muñoz et al., 2016; Álvarez-Hernán et al., 2019) and new-borns of altricial mammals (Palatroni et al., 1990). In contrast, newly hatched individuals of *C. coturnix* present a functional visual system with a totally differentiated retina in which mitotic activity is almost absent, as has been previously shown in the precocial retina of *G. gallus* (Fischer and Reh, 2000; Álvarez-Hernán et al., 2021a). Similar results have been described in the retina of precocial fish (Candal et al., 2005; Ferreiro-Galve et al., 2010; Bejarano-Escobar et al., 2012; Álvarez-Hernán et al., 2019) and mammals (Loeliger and Rees, 2005).

Comparative analyses of cell proliferation in the telencephalon of altricial and precocial birds have shown delayed telencephalic neurogenesis in the altricial species (DeWulf and Bottjer, 2005; Striedter and Charvet, 2008; Charvet and Striedter, 2009a,b, 2011). In these studies, researchers found that the major period of telencephalic neurogenesis ends approximately 1 week after hatching, although residual neurogenesis persists into adulthood in altricial birds. However, the major period of generation of new neurons is completed by hatching in precocial species such as quail and chicken (Nikolakopoulou et al., 2006; Striedter and Charvet, 2008).

Therefore, the present study reveals that the *T. guttata* retina exhibits a lower maturation status at hatching than *C. coturnix*, indicating higher prenatal neurogenesis and retinal maturation in precocial bird species.

### The Progression of Apical and Non-apical Mitosis in the Bird Retina

During retinogenesis, the nucleus of the retinal progenitors oscillates from the apical to the basal surface in proliferative neuroepithelia. The movement of the nucleus is in phase with the cell cycle, and M phase always occurs at the apical surface of the neuroepithelium (Baye and Link, 2007). Retinal progenitors either divide symmetrically to generate other progenitors or divide asymmetrically to generate both a progenitor and a postmitotic migrating neuroblast (Xiang, 2013). However, not all retinal progenitor divisions are restricted to the apical surface of the neuroepithelium, and committed horizontal cell progenitors divide in non-apical regions in the retina of fish (Godinho et al., 2007; Weber et al., 2014) and birds (Edqvist and Hallböök, 2004; Boije et al., 2009, 2016). Therefore, the pattern of cessation of neurogenesis could be monitored by the disappearance of mitotic figures in the retinal tissue (Sharma and Ehinger, 1997; Barton and Levine, 2008; Bejarano-Escobar et al., 2012).

In the present study we have shown that mitotic activity is abundant in the altricial retina even at hatching stages (see above). However, mitotic activity almost disappears from the apical surface in the entire quail retina at E10. Similar results were found when monitoring the disappearance of non-apical cell divisions. By these embryonic stages, the quail retina is fully differentiated and the main retinal cell types could be characterized immunohistochemically (unpublished observations, Javier Francisco-Morcillo). Then, our findings are in line with previous studies showing that neurogenesis ceases at embryonic stages in the precocial bird retina, and hatchlings present a mature and functional visual system (Fischer and Reh, 2000; Álvarez-Hernán et al., 2021a).

It has been described that during ontogeny, many morphological features begin earlier in the precocial bird species than in the altricial ones (Murray et al., 2013). In the case of the developing visual system, the formation of the optic cup, the formation of the optic vesicle, the onset of differentiation of ganglion cells and photoreceptors, and the emergence of the plexiform layers occur earlier in *G. gallus* than in *T. guttata* (Álvarez-Hernán et al., 2018, 2020). Here we also showed that the onset of the appearance of non-apical mitoses in *T. guttata* take place at E6 (St30), while in *C. coturnix* non-apical mitoses were numerous at E5 (St27). Furthermore, the density of non-apical mitosis in the central region of the *T. guttata* retina reached a peak at E8-9, whereas the highest density values were found by E7.5-8 in *C. coturnix* (present study) and in *G. gallus* (Boije et al., 2009). Therefore, the onset of appearance of non-apical mitoses occurs at later stages in the zebra finch than in the quail.

Non-apical mitoses have been linked to horizontal cell differentiation in fish (Godinho et al., 2007; Weber et al., 2014; Engerer et al., 2017) and birds (Edqvist and Hallböök, 2004; Boije et al., 2009, 2016; Shirazi Fard et al., 2013, Shirazi Fard et al., 2014a,b). We used specific markers of horizontal cells to establish a possible relationship between non-apical mitosis and horizontal cell differentiation. Prox1 is considered a pan-marker of horizontal cells, whereas the transcription factor Isl1 is expressed by the axon-less horizontal cell subtype (Edqvist et al., 2008; Boije et al., 2009). A similar staining to that described by those authors in the chicken retina has been detected in the *T. guttata* (Álvarez-Hernán et al., 2020) and in the *C. coturnix* retinal tissue (present study). Although we never detected co-labeling of these horizontal cell markers with non-apical pHisH3 mitoses, there was a close spatiotemporal relationship between the onset of the appearance of non-apical pHisH3-immunoreactive mitoses and the onset of Prox1-expression in the different retinal sectors, in concordance with the results obtained in the chicken (Edqvist et al., 2008; Boije et al., 2009). These findings suggest a possible relationship between the appearance of non-apical mitoses and horizontal cell differentiation in the retina of both bird species. Again, these results may suggest that the onset of horizontal cell differentiation is delayed in the altricial bird retina.

In conclusion, the present study provides comprehensive data on distinct patterns of retinal development between the altricial zebra finch and the precocial Japanese quail, which may serve as empirical reference in future studies. While the order of different events involved in retinal maturation is the same during development, their specific timing differs between the altricial and precocial bird species. The retina of the precocial birds exhibits higher degree of maturation at hatching, thus providing evidence for the notions that precocial species might have acquired the morphological machinery required to attain their higher functional state at hatching. Together, our results expand our current understanding of the timing and cellular differences that regulate patterns of avian retinal growth and maturation, and provides a better understanding of the evolution of avian altriciality and precociality.

## Data Availability Statement

The original contributions presented in the study are included in the article/Supplementary Material, further inquiries can be directed to the corresponding author/s.

## Ethics Statement

The animal study was reviewed and approved by Comité de Ética en Experimentación Animal.

## Author Contributions

JF-M, GM-P, and JR-L contributed to conception and design of the study. JM-R, AM, and YG organized the database. GM-P performed the statistical analysis. JF-M wrote the first draft of the manuscript. GÁ-H wrote sections of the manuscript and performed histological and immunohistochemical analysis. AA performed the immunohistochemical analysis. All authors contributed to manuscript revision, read, and approved the submitted version.

## Conflict of Interest

The authors declare that the research was conducted in the absence of any commercial or financial relationships that could be construed as a potential conflict of interest.

## Publisher’s Note

All claims expressed in this article are solely those of the authors and do not necessarily represent those of their affiliated organizations, or those of the publisher, the editors and the reviewers. Any product that may be evaluated in this article, or claim that may be made by its manufacturer, is not guaranteed or endorsed by the publisher.

## Footnotes

1
http://rsb.info.nih.gov/ij/

## Supplementary Material

Supplementary Figure 1 Toluidine blue-stained semi-thin section **(A)** and expression of cell markers in cryosections **(B–H)** of the embryonic retina of *C. coturnix*. Cryosections were counterstained with DAPI. **(A,A′)** Mitotic figures were found in the apical surface (arrows) and in non-apical regions [arrowheads in panels **(A,A′)**]. **(B)** Apical (arrows) and non-apical (arrowhead) pHisH3-immunoreactive mitoses were found in the NbL at St34. **(C)** Prox1-immunoreactive presumptive horizontal cells (arrows) were found in the horizontal cell layer at St35. Prox1 immunoreactivity was also found in the nuclei of migrating precursors of horizontal cells in more internal regions of the NbL (arrowheads). **(D)** Prox1-immunolabelled horizontal cells (arrows) were mainly located in the laminated retina at St42. Prox1-immunostained horizontal cell precursors were found in more internal regions (arrowheads). A subpopulation of amacrine cells was immunopositive against Prox1 antibody (double arrowheads). **(E)** At St35, Isl1 immunosignal was detected in presumptive horizontal cells (arrows) and migratory neuroblasts (arrowheads) in the undifferentiated retina. **(F)** At St35 Is1 immunoreactivity was found in the nuclei of subpopulations of ganglion, amacrine, bipolar and horizontal cells. **(G,H)** Prox1 immunoreactivity was found in horizontal cells at St 42 (arrowheads) and a sub-population of Prox1 immunoreactive horizontal cells was co-labeled for Isl1 (double arrowheads). *Abbreviations: GCL, ganglion cell layer; INL, inner nuclear layer; IPL, inner plexiform layer; NbL, Neuroblastic layer; ONL, outer nuclear layer. Scale bars:* 50 μm in A-G; 5 μm in panel **(A′)**; 25 μm in panel **(H)**.
