# Supplementary figures and images for: Timing and Distribution of Mitotic Activity in the Retina During Precocial and Altricial Modes of Avian Development
Source: Front Neurosci. 2022 May 9;16:853544. doi: 10.3389/fnins.2022.853544 (PMC9125163; doi:10.3389/fnins.2022.853544)

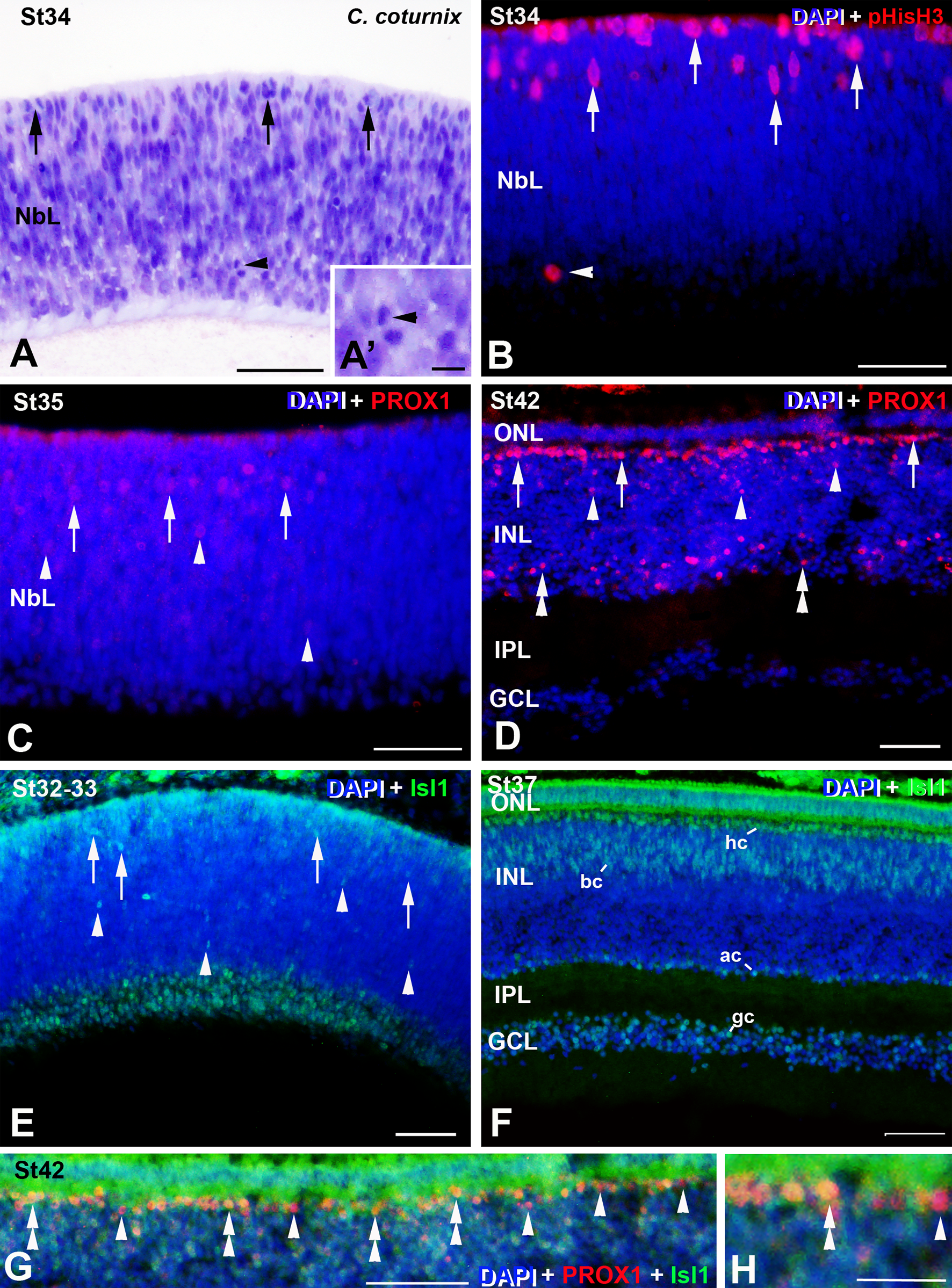

Supplement: Supplementary Figure 1 — Toluidine blue-stained semi-thin section (A) and expression of cell markers in cryosections (B–H) of the embryonic retina of C. coturnix. Cryosections were counterstained with DAPI. (A,A′) Mitotic figures were found in the apical surface (arrows) and in non-apical regions [arrowheads in panels (A,A′)]. (B) Apical (arrows) and non-apical (arrowhead) pHisH3-immunoreactive mitoses were found in the NbL at St34. (C) Prox1-immunoreactive presumptive horizontal cells (arrows) were found in the horizontal cell layer at St35. Prox1 immunoreactivity was also found in the nuclei of migrating precursors of horizontal cells in more internal regions of the NbL (arrowheads). (D) Prox1-immunolabelled horizontal cells (arrows) were mainly located in the laminated retina at St42. Prox1-immunostained horizontal cell precursors were found in more internal regions (arrowheads). A subpopulation of amacrine cells was immunopositive against Prox1 antibody (double arrowheads). (E) At St35, Isl1 immunosignal was detected in presumptive horizontal cells (arrows) and migratory neuroblasts (arrowheads) in the undifferentiated retina. (F) At St35 Is1 immunoreactivity was found in the nuclei of subpopulations of ganglion, amacrine, bipolar and horizontal cells. (G,H) Prox1 immunoreactivity was found in horizontal cells at St 42 (arrowheads) and a sub-population of Prox1 immunoreactive horizontal cells was co-labeled for Isl1 (double arrowheads). Abbreviations: GCL, ganglion cell layer; INL, inner nuclear layer; IPL, inner plexiform layer; NbL, Neuroblastic layer; ONL, outer nuclear layer. Scale bars: 50 μm in A-G; 5 μm in panel (A′); 25 μm in panel (H). [file Image_1.TIF]
